# Necessity of Assessing Biological Exposure to Arsenic Species by Two Representative Analytical Methods

**DOI:** 10.3390/toxics9060138

**Published:** 2021-06-11

**Authors:** Jeong-Wook Seo, Young-Seoub Hong

**Affiliations:** 1 Environmental Health Center, Dong-A University, Busan 49201, Korea; jwseo@dau.ac.kr; 2 Department of Preventive Medicine, Dong-A University, Busan 49201, Korea

**Keywords:** arsenate, arsenite, atomic absorption spectroscopy, biological exposure assessment, dimethylarsenic acid, inductively coupled plasma mass spectrometry, monomethylarsinic acid

## Abstract

Arsenic (As) exists as highly toxic chemical species. Chronic exposure to its inorganic form can cause multiple organ failure and skin cancer in humans, warranting the need to determine the toxicity of each chemical species. This study evaluated the proportions of exposure to four chemical species of As (cAs), namely arsenite (AsIII), arsenate (AsV), monomethylarsinic acid (MMA), and dimethylarsenic acid (DMA), and it confirmed the necessity of evaluating biological exposure to cAs. Urine samples were collected from 457 subjects residing near 103 abandoned metal mines. Hydride generation atomic absorption spectroscopy (HG-AAS) was performed to measure the combined concentration of four cAs (hAs_AAS_). High-performance liquid chromatography and inductively coupled plasma-mass spectrometry (HPLC-ICP-MS) were performed to determine the concentrations of the individual cAs and the sum of the four cAs (hAs_ICP_). The proportions of AsV and MMA were relatively higher in the low-hAs_ICP_ concentration section. These findings suggest that hAs_AAS_, which is mainly used for its cost-efficiency, is limited for evaluating exposure. Though hAs_AAS_ was found to exist in a low concentration, highly toxic AsV and MMA could be observed in high concentrations. Therefore, HPLC-ICP-MS is recommended for assessing cAs in environmentally vulnerable areas such as abandoned metal mines.

## 1. Introduction

In the environment, As occurs in various oxidation states and in both inorganic and organic forms. The chemical species of arsenic (cAs) differ in terms of their toxicity and health effects. In general, inorganic arsenic (iAs) is more toxic than organic arsenic (oAs), and arsenite (AsIII) is more toxic than arsenate (AsV) [1]. A previous study reported that oAs in methylated metabolites such as monomethylarsinic acid (MMA) and dimethylarsenic acid (DMA) are nontoxic [2]. In contrast, other studies have suggested that monomethylarsonous acid (MMA (III)) is highly toxic [3,4]. Chronic exposure to iAs affects not only the skin but also the respiratory, gastrointestinal, cardiovascular, nervous, hepatic, endocrine, and hematopoietic systems [5]. Additionally, long-term iAs exposure may induce skin cancer [2].

Exposure to most As compounds occurs through drinking water and food intake [1]. Exposure may be a consequence of the natural occurrence of highly concentrated As in groundwater. Food is the second leading source of As intake [6]. Exposure might result from the consumption of crops grown in As-contaminated soils and dust inhalation in polluted areas such as mines [1,7]. Occupational exposure can occur through the inhalation of dust or aerosols with high As levels [8,9].

Human As exposure has been assessed by measuring total arsenic (tAs) using inductively coupled plasma-mass spectrometry (ICP-MS) and/or hazardous arsenic species (hAs: inorganic-related As species—AsIII, AsV, MMA, and DMA) using hydride generation atomic absorption spectroscopy (HG-AAS). However, the importance of knowing the effects of cAs on health has been emphasized, and the assessment of exposure to the individual species is required. Korea’s Environmental Health Act stipulates the duty of the epidemiological investigation and management in areas where environmental pollution is a concern. The Health Effects Survey of Abandoned Metal Mines (AMS: Abandoned Metal Mines Survey) has been carried out since 1996 as biomonitoring for abandoned metal mines, which are areas of concern for representative environmental pollution. AMS mainly evaluates human exposure to hazardous metals from abandoned metal mines, and individual follow-up management is carried out for subjects exposed to high concentrations. This includes the assessment of arsenic exposure due to contaminated drinking water and soil. Through the AMS, the concentration of hAs measured by HG-AAS (hAs_AAS_) was evaluated. In addition, AsIII, AsV, MMA, and DMA were quantitatively analyzed only when the hAs_AAS_ was high using high-performance liquid chromatography and inductively coupled plasma-mass spectrometry (HPLC-ICP-MS), which required a high cost, and their sum, hAs_ICP_, was evaluated.

In this study, we aimed to confirm the necessity of evaluating biological exposure to cAs. For this purpose, AMS data were used to confirm the characteristics and proportion of the exposure level to cAs. We also discussed the consistency and interchangeability of hAs_AAS_ and hAs_ICP_ with AsIII, AsV, MMA, and DMA depending on the hAs_AAS_ concentration.

## 2. Materials and Methods

### 2.1. Study Population

**Figure 1:**
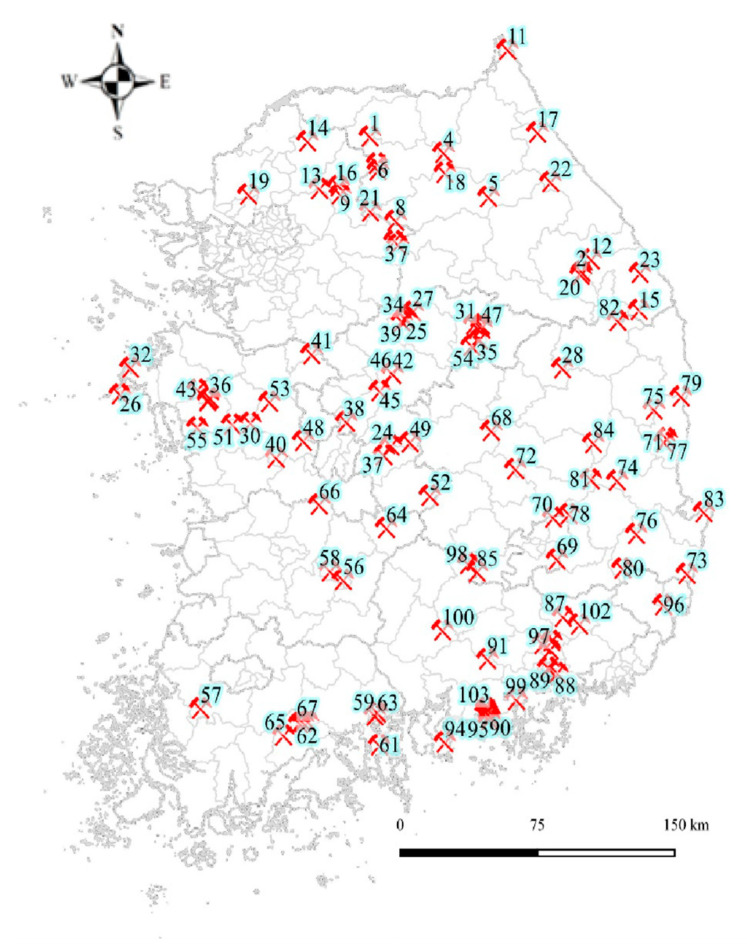
Location of the 103 abandoned metal mines.

The Korean Ministry of the Environment (KMOE) reported data for general and specific investigations on the soil and water pollution levels of 857 abandoned metal mines during 1996–2005. A preliminary investigation was conducted on 358 abandoned metal mines with known risks of inducing health effects. The overall risk was quantified according to the results of the environmental pollution and impact assessments. First-level (08–11) and second-level (13–17) abandoned metal mines surveys (AMSs) were performed in the top 10% of 38 regions and in the top 10–40% of 103 mines, respectively (Figure 1). In the present study, second-level AMS data were used. A total of 4500 people were surveyed over 5 years in five regions, and standardized tools and guidelines were used. A questionnaire on subject residential areas and characteristics was conducted, and analyses of harmful metals, such as lead and cadmium in the blood and As in the urine, were performed. The hAs_AAS_ concentrations were evaluated in all 4500 people. The top 10% or those with >100 µg/L were defined as subjects with a high As exposure. In these subjects, cAs was measured using HPLC-ICP-MS. There were 457 subjects whose creatinine levels were within the normal range of 0.3–3.0 g/L, and they were selected as the final subjects for analysis [10,11].

### 2.2. Sample Collection

All procedures related to biological sample collection and heavy metal analysis were performed in accordance with the guidelines of the Korea National Institute of Environmental Research (KNIER) [12,13]. To prevent contamination, urine samples were collected only after the subjects had become familiar with the collection method. Ten milliliters of midstream urine was collected in a dedicated urine cup (PLC-03701 natural polypropylene jar with a 58–400 white polypropylene unlined cap 120 m; Qorpak, Bridgeville, PA, USA). The initial urine stream was discarded. Five-milliliter aliquots of the collected urine were placed in dedicated containers (229412 centrifuge tube, 15 mL, polypropylene, CELLTREAT, Pepperell, MA, USA) and stored at −80 °C.

### 2.3. Arsenic Determined Using Hydride Generation Atomic Absorption Spectroscopy

HG-AAS (inAAcle 900Z atomic absorption spectrometer/FIAS 100 flow injection for atomic spectroscopy system, PerkinElmer, Waltham, MA, USA) was used for quantitative hAs analysis. As a pre-reductant required for analysis, 10% L-Cysteine (Sigma-Aldrich Corp., St. Louis, MO, USA) was used in 0.03 M HCl, and 0.5% NaBH_4_ in 0.05% NaOH was used as a hydride reagent. Additionally, 0.03 M HCl was used as the carrier solution. Impurities were removed from the urine samples using a 0.22-µm filter. The sample was diluted 10 times to 10 mL and measured after a 1:1 reaction with pre-reductant. Standard stock solution (Inorganic ventures, Christiansburg, VA, USA) samples were used to plot calibration curves (Inorganic ventures, Christiansburg, VA, USA). Standard materials (ClinChek urine control lyophilized for trace elements, Levels I and II, RECIPE Chemicals, Dessauerstraße, München, Germany) were used to confirm process reliability. The analysis library was accredited by the KNIER Quality Assurance Program.

### 2.4. Arsenic Speciation Determined Using High-Performance Liquid Chromatography with Inductively Coupled Plasma-Mass Spectrometry

To separate and quantify cAs, impurities were removed from urine samples using a 0.22-µm filter, and the samples were then diluted with an appropriate amount of dextrose solution. As a mobile phase, 10 mM ammonium carbonate (99.99%), 10 mM Trizma^®^ base (99.9%) (Sigma-Aldrich Corp., St. Louis, MO, USA), and 15 mM ammonium sulfate (99.5%) (Junsei chemical Co., Ltd., Chuo-ku, Tokyo, Japan) were used. HPLC-ICP-MS (Agilent Technologies 1260 series high-performance liquid chromatography/7700 series inductively coupled plasma-mass spectrometer, Agilent Technologies, Santa Clara, CA, USA) fitted with a Hamilton PRP X-100 column was used to quantitate AsIII, AsV, MMA, and DMA. Calibration curves were plotted using standard AsIII, AsV, MMA, and DMA (Sigma-Aldrich Corp., St. Louis, MO, USA), and their accuracy was verified using two reference standards (Standard Reference Material No. 2669, National Institute of Standards and Technology (NIST), Gaithersburg, MD, USA; Certified Reference Material No. 18, National Institute for Environmental Studies, Onogawa, Tsukuba, Japan). The analytical laboratory was accredited by the quality assurance program of the German External Quality Assessment Scheme (G-EQUAS) operated by Friedrich-Alexander University, Erlangen-Nuremberg, Germany [14].

### 2.5. Statistical Analysis

All data were analyzed using SASv. 9.4 (SAS Institute, Cary, NC, USA). Means and dispersion were estimated to confirm the distribution of urinary arsenic concentration (UAL: urine arsenic level) determined using HG-AAS and HPLC-ICP-MS. The concordance correlation coefficient (CCC) was calculated to evaluate consistency between the methods. For the proportion of cAs measured by HPLC-ICP-MS, the least square means (LSMs) were calculated after adjusting for the subject characteristics. A simple nonlinear model was used to estimate the proportions of cAs according to the UAL returned by HG-AAS. Goodness-of-fit was determined for the observed values. The significance level was set at below 5% for all statistics.

### 2.6. Ethics Statement

Participants were given fully explanations of the study purpose and procedure, and an agreement for the questionnaire and blood sample collections was secured from them. Personal information and specimen analyses were then provided to all participants. The present study was approved by the Chung-Ang University Institutional Review Board (IRB No. 1041078-201805-HRBR-103-01, approved on 26 June 2018).

## 3. Results

### 3.1. Distribution of Urinary Arsenic Concentration

Table 1 shows the UAL distribution of 457 samples analyzed using HG-AAS and HPLC-ICP-MS. One of the 103 abandoned metal mines showed very high As concentrations in potable tap water and groundwater. Hence, this area introduced an extreme value to the overall distribution, and the data were stratified for the estimation. The geometric means (GMs) (95% confidence interval (CI)) of the UAL for each of the 102 mines were 0.12 (0.10–0.14), 0.34 (0.29–0.39), 0.92 (0.76–1.12), and 70.61 (65.78–75.81) µg/g-creatinine (µg/g-cr) for AsIII, AsV, MMA, and DMA, respectively. The UAL of hAs_ICP_ or the sum of the four cAs associated with iAs (UAL_ICP_) was 77.05 (72.11–82.32) µg/g-cr. In contrast, the UAL of hAs_AAS_ (UAL_AAS_) was 55.14 (50.76–59.91) µg/g-cr. The single mine with As-contaminated drinking water showed 16.12 (6.12–42.46) and 5.30 (1.83–15.36) µg/g-cr for AsIII and AsV, respectively. The sum of AsIII and AsV was 31.44 (21.44–46.12) µg/g-cr, which was 50 times higher than the sum of that of the other 102 mines (0.65 (0.56–0.75) µg/g-cr). The values for MMA (20.06 (12.04–33.43) µg/g-cr) and DMA (183.60 (146.52–230.07) µg/g-cr) were ~30 times higher than the sum of those in the 102 mines. The arithmetic mean (AM) ± standard deviation (SD) of the proportion of chemical species was the highest for DMA (91.74 ± 9.67%), followed by MMA (4.24 ± 4.40%), AsV (2.82 ± 6.43%), and AsIII (1.21 ± 2.89%).

### 3.2. Agreement Between HG-AAS and HPLC-ICP-MS Arsenic Concentration Measurements

**Figure 2:**
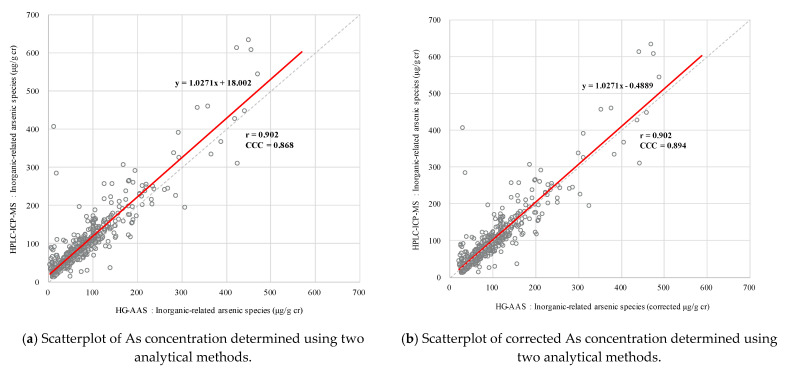
Scatterplots of As concentrations determined using HG-AAS and HPLC-ICP-MS in 103 abandoned metal mines.

Scatterplots and correlation coefficients were calculated to assess the correlation between HG-AAS and HPLC-ICP-MS in terms of UAL. The scatterplots showed a positive trend. The Pearson’s correlation coefficient (r) and CCC were 0.902 and 0.868, respectively (Figure 2a). When UAL_ICP_ was an independent variable, the estimated simple linear regression model was UAL_ICP_ = 18.00 + 1.03 × (UAL_AAS_) and the slope of the regression was near unity and increased to the same level. However, the y-intercept was 18.00. Therefore, the average UAL_ICP_ for each observation was 18 µg/g-cr higher than that of UAL_AAS_. UAL_AAS_ = UAL_AAS_ + 18.00 was corrected to fit the reference line UAL_ICP_ = 1.00 × (UAL_AAS_). The CCC was adjusted to 0.894, and the estimated regression equation was UAL_ICP_ = −0.49 ± 1.03 × (corrected UAL_AAS_) (Figure 2b).

### 3.3. Proportions of Arsenic Species Concentrations

**Figure 3:**
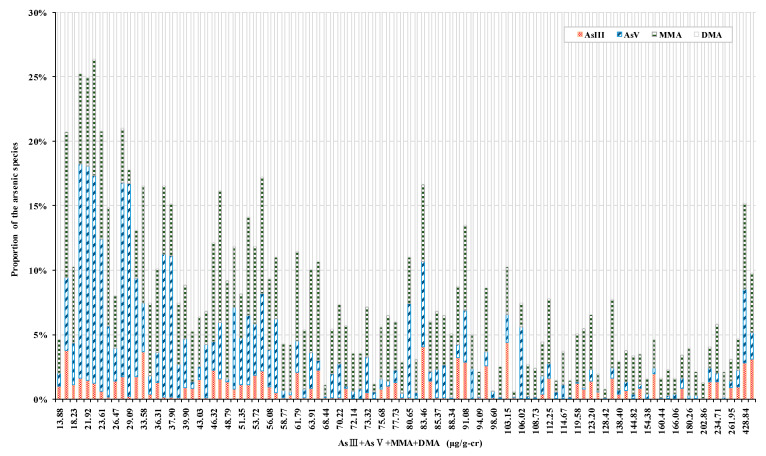
Proportion of As species relative to the sum of concentrations of four As species in 102 abandoned metal mines. Estimation of proportion of cAs using the simple nonlinear model (proportion of AsIII (%)) = 2.724 × (1/(hAs^0.529^)), R^2^ = 0.104; (proportion of AsV (%)) = 0.0000998 × hAs^2^ − 0.000603 × hAs + 7.114, adjusted R^2^ = 0.273; (proportion of MMA (%)) = 0.0000645 × hAs^2^ − 0.0381 × hAs + 6.773, adjusted R^2^ = 0.306; (proportion of DMA (%)) = −0.000184 × hAs^2^ + 0.106 × hAs + 84.716, adjusted R^2^ = 0.318. Goodness-of-fit between cAs estimated by projecting hAs_AAS_ on the simple nonlinear model and actual observed cAs. Observed AsIII = 0.424 × (estimated AsIII) − 0.109, R^2^ = 0.0254; observed AsV = −0.485 × (estimated AsV) + 2.413, R^2^ = 0.0233; observed MMA = 1.085 × (estimated MMA) − 0.00760, R^2^ = 0.0432; observed DMA = 1.121 × (estimated DMA) − 7.165, R^2^ = 0.843.

The cAs proportions (95% CI) were estimated by adjusting for sex, age, period of residence, drinking water, smoking status, and drinking status and compared to UAL_ICP_. Those of AsIII, AsV, MMA, and DMA were 0.79 (0.36–1.22)%, 2.58 (1.62–3.53)%, 4.33 (3.67–4.98)%, and 92.31 (90.90–93.71)%, respectively. The proportions of AsIII, AsV, iAs, MMA, and AsIII, AsV, and MMA significantly increased with the numbers of males, children, and young adults, period of residence, no seafood intake in the last week, and decreasing distance from a mine. UAL_ICP_ was divided by concentration into four quartiles (Q1–Q4). AsIII had a significantly higher concentration in Q4 than the others. In contrast, AsV and MMA had significantly higher concentrations in Q1 than the others (Table 2). To estimate cAs according to the UAL_AAS_, the proportion of the average cAs for each percentile section of UAL_ICP_ was used. A nonlinear model (proportion of cAs = f (UAL_ICP_) + ε) was estimated for all four cAs and served as the coefficient of determination (R^2^). Assuming consistency between UAL_ICP_ and UAL_AAS_, the UAL for AsIII, AsV, and MMA was estimated by applying the model equation chosen according to the observed UAL_AAS_. DMA was evaluated according to the difference between UAL_AAS_ and the estimated AsIII, AsV, and MMA. To establish the goodness-of-fit of the cAs estimation model, we assessed the R^2^ of the estimate for the actual observed cAs value. However, none of the values for AsIII, AsV, and MMA were satisfactory (Figure 3).

## 4. Discussion

Here, we used AMS data to show the characteristics of the cAs distribution and composition ratios in residents located near these sites. We observed consistency between the individual exposure assessment data determined using HPLC-ICP-MS and the hAs determined using HG-AAS.

The gold standard technique for evaluating As exposure is HPLC-hydride generation-coupled to inductively coupled plasma-mass spectrometry (HPLC-HG-ICP-MS). However, HG-AAS is a cost-effective alternative method for quantifying hAs, namely the sum of AsIII, AsV, MMA, and DMA [15]. This metric is used for As biomonitoring in Korea. Nevertheless, iAs toxicity differs from that of oAs. The cAs must be evaluated in areas where there is a high risk of iAs exposure. In the present study, the adjusted GM of the UAL of 102 mines in the AMS were 0.10, 0.31, 0.90, and 68.75 µg/g-cr for AsIII, AsV, MMA, and DMA, respectively (Table S1). The 2015–2016 U.S. National Health and Nutrition Examination Survey (NHANES) showed that AsIII, AsV, and MMA were below their limits of detection (LOD) in 60% of all subjects and DMA was only 3.41 µg/g-cr [16]. According to the 2008–2009 health statistics of the Korea National Health and Nutrition Examination Survey (KNHANES), the representative values of AsIII, AsV, MMA, and DMA in the general Korean population were 0.018, 0.005, 0.020, and 11.013 µg/g-cr, respectively [17]. Both the general population and those dwelling in vulnerable areas are exposed to iAs. The Korean population has the highest seafood consumption per capita [18,19] and a relatively high DMA level. DMA occurs in certain algae and shellfish whose consumption may increase urinary DMA [20,21]. The iAs are ~100 times more toxic than oAs. Among the iAs, AsIII is ~60 times more toxic than AsV [1]. In regions with a high seafood intake, the evaluation of human exposure via tAs and hAs artificially increases DMA. Hence, actual human exposure and health risk might be either exaggerated or underestimated, and human exposure should be evaluated through cAs.

UAL_ICP_ was ~18 µg/g-cr higher than UAL_AAS_. The estimate for the slope of the regression was ~1.03; the r and CCC were 0.902 and 0.868, respectively; and the CCC of the corrected UAL_AAS_ was 0.89. Bühl et al. [15] showed consistency between the output of HG-AAS and HPLC-HG-ICP-MS for 40 samples. Their r value was 0.971, and their estimated regression model was UAL_ICP_ = 0.59 + 0.94 × (UAL_AAS_). Lindberg et al. [22] reported a regression model of UAL_ICP_ = −5.19 + 1.02 × (UAL_AAS_) for 89 samples. Relative to UAL_ICP_ = −0.49 + 1.03 × (corrected UAL_AAS_) for the current study, it differed in terms of sample size and UAL range. However, the slopes were similar for both models. Therefore, high consistency and reliability may be achieved by adjusting for concentration differences between analytical methods.

The proportion of each cAs relative to UAL_ICP_ was used to assess exposure trends. The proportion of DMA was 92.31%, and the coefficient of the determination of DMA for UAL_ICP_ was 0.958. Thus, UAL_ICP_ was strongly influenced by DMA. In contrast, high UAL_ICP_ concentrations were not indicative of high AsV or MMA concentrations. Moreover, relatively high AsV and MMA concentrations were measured at low UAL_ICP_ concentrations (Figure S1). The correlation coefficients of UAL_ICP_ with AsV and MMA were −0.172 and −0.071, respectively. Thus, they had an inverse linear relationship (Table S2). There was consistency between UAL_ICP_ and UAL_AAS_. For this reason, high AsV and MMA concentrations were not necessarily associated with high UAL_AAS_ concentrations. KNIER [23] reported that HPLC-ICP-MS has low LOD and high analytical sensitivity and can detect lower element concentrations using fewer biological samples than HG-AAS. Furthermore, HPLC-ICP-MS can conduct multi-element analyses. However, cAs analysis is not cost-effective for large-scale biomonitoring. Consequently, UAL_AAS_ has been used to select high-concentration exposure in AMS and to evaluate cAs [12]. However, the present study showed that UAL_AAS_ may have limited efficacy at screening for exposure to high iAs concentrations originating from environmental pollution sources. Therefore, exposure assessment through cAs, rather than UAL_AAS_, is recommended for vulnerable areas where seafood consumption and human exposure risks of iAs are high. In particular, recently, a study was published that found a high possibility of As contamination on agricultural soils in abandoned metal mines in Korea [7]. This study assessed the interchangeability of cAs based on UAL_AAS_ and established the unreliability of the estimated exponential and nonlinear second-order polynomial models. In other words, there is a tendency toward the UAL of cAs and the proportion of cAs according to UAL_ICP_. However, the estimated model introduces substantial calibration errors. UAL_ICP_ could not isolate the causes for the change in highly toxic AsIII concentration. Therefore, cAs must be evaluated for vulnerable areas.

In the present study, exposure to relatively high UAL_AAS_ concentrations was determined for residents living adjacent to 103 abandoned metal mine areas known to pose environmental and human health risks. We compared and evaluated human As exposure level from large-scale data and analyzed cAs. Here, we used large-scale data to simultaneously analyze both UAL_AAS_ and cAs in 457 Korean samples. However, only those exposed to high UAL_AAS_ concentrations in specific vulnerable areas were evaluated. For this reason, the validity of extrapolating these results to the general population is questionable. Evaluation agreement showed that the UAL_ICP_ level was higher than that of UAL_AAS_. Moreover, the minimum value of >18 µg/g-cr obtained for the corrected UAL_AAS_ was also a limitation. Nevertheless, the present study effectively assessed the consistency between two representative analytical methods for human As exposure and underscored the importance of evaluating cAs in vulnerable areas.

## 5. Conclusions

Chronic exposure to low As concentrations was established for the contaminated areas studied here. A few areas presented with a risk of exposure to high As concentrations caused by polluted drinking water. However, as the Korean population has a high per capita seafood intake, the DMA exposure risk is also elevated. The evaluation of human exposure through tAs and hAs is associated with a high probability of exaggeration or underestimation because of the toxicity of these cAs. Hence, it is advised to assess exposure to highly toxic iAs through cAs. For this reason, cAs should be quantitated using HPLC-ICP-MS. There is reliable consistency between UAL_ICP_ and UAL_AAS_. A viable alternative is the estimation of iAs through interchangeable cAs and cost-effective AAS.

## Figures and Tables

**Table 1:** Distribution of urinary arsenic concentration in residents living near abandoned metal mines.

Group	Device	Urinary As Species		Mean ± SD	GM (95% CI)	Median (Range)	P75	P90	P95	P99
102 mines (*n* = 443) (μg/g-cr)	HPLC -ICP -MS	Inorganic As	AsIII	0.97 ± 4.65	0.12 (0.10–0.14)	<LOD (<LOD–74.41)	<LOD	2.04	4.43	12.84
			AsV	1.70 ± 4.87	0.34 (0.29–0.39)	0.12 (<LOD–62.85)	1.37	4.40	7.33	22.22
			Subtotal	2.66 ± 8.34	0.65 (0.56–0.75)	0.32 (<LOD–122.92)	2.37	6.48	9.59	26.63
		Organic As	MMA	3.41 ± 6.88	0.92 (0.76–1.12)	2.33 (<LOD–99.79)	4.29	6.78	9.45	28.72
			DMA	92.98 ± 77.27	70.61 (65.78–75.81)	73.65 (9.37–622.79)	115.11	171.38	234.77	390.90
			Subtotal	96.39 ± 79.41	73.94 (68.99–79.24)	77.40 (9.47–633.75)	118.34	171.64	238.00	456.52
		AsIII, AsV, MMA, and DMA		99.05 ± 81.96	77.05 (72.11–82.32)	80.17 (12.60–633.97)	120.28	173.99	239.10	460.76
	HG-AAS			77.43 ± 66.27	55.14 (50.76–59.91)	63.29 (2.69–469.43)	101.21	150.06	190.17	357.50
high Level 1 mine (*n* = 14) (μg/g-cr)	HPLC -ICP -MS	Inorganic As	AsIII	27.37 ± 18.90	16.12 (6.12–42.46)	27.63 (0.07–66.06)	37.71	56.15	66.06	66.06
			AsV	10.50 ± 7.30	5.30 (1.83–15.36)	8.93 (0.08–22.41)	17.32	18.77	22.41	22.41
			Subtotal	37.87 ± 23.19	31.44 (21.44–46.12)	31.31 (7.33–88.46)	50.77	74.52	88.46	88.46
		Organic As	MMA	26.92 ± 18.52	20.06 (12.04–33.43)	24.41 (3.82–60.95)	38.82	58.06	60.95	60.95
			DMA	196.19 ± 70.13	183.60 (146.52–230.07)	173.70 (87.36–298.77)	263.13	294.71	298.77	298.77
			Subtotal	223.11 ± 85.25	206.87 (162.51–263.33)	209.73 (91.18–359.72)	292.98	352.77	359.72	359.72
		AsIII, AsV, MMA, and DMA		260.98 ± 104.69	240.70 (187.70–308.66)	243.50 (98.50–448.18)	335.27	427.29	448.18	448.18
	HG-AAS			283.31 ± 110.07	260.67 (201.39–337.40)	264.31 (98.08–439.90)	386.78	424.70	439.90	439.90
Proportion of each As species Relative to sum of concentrations of 4 As species in 103 mines (*n* = 457), %	HPLC -ICP -MS	Inorganic As	AsIII	1.21 ± 2.89	0.17 (0.15–0.20)	0.11 (0.01–22.00)	0.23	4.93	6.95	14.14
			AsV	2.82 ± 6.43	0.46 (0.38–0.55)	0.24 (0.01–52.48)	2.22	8.85	13.75	31.75
			Subtotal	4.03 ± 7.20	0.92 (0.78–1.09)	0.73 (0.03–52.66)	4.76	12.75	19.74	31.97
		Organic As	MMA	4.24 ± 4.40	1.27 (1.04–1.55)	3.26 (0.01–23.33)	6.59	10.48	13.45	17.35
			DMA	91.74 ± 9.67	91.14 (90.14–92.15)	95.28 (45.95–99.96)	98.86	99.76	99.84	99.92
			Subtotal	95.97 ± 7.20	95.64 (94.88–96.42)	99.27 (47.34–99.97)	99.82	99.88	99.91	99.95
		AsIII, AsV, and MMA		8.26 ± 9.67	3.32 (2.86–3.86)	4.72 (0.04–54.05)	11.24	22.52	28.11	43.14

LOD: limit of detection; substitute LOD/2 value for all. LOD of AsIII = 0.100; AsV = 0.164; MMA = 0.078; DMA = 0.022. Urinary creatinine reference range: 0.3–3.0 g/L.

**Table 2:** Adjusted proportions of as species concentration relative to the sum of the concentrations of 4 As species in 103 abandoned metal mines.

	*n* (%)	Adjusted Proportion (95% CI) (%)													
		Inorganic As						Organic As						AsIII, AsV, and MMA	
		AsIII		AsV		Subtotal		MMA		DMA		Subtotal			
Total	456 (100.0)	0.79 (0.36–1.22)		2.58 (1.62–3.53)		3.32 (2.28–4.36)		4.33 (3.67–4.98)		92.31 (90.90–93.71)		96.68 (95.64–97.72)		7.69 (6.29–9.10)	
Sex															
Male	187 (41.0)	1.39 (0.89–1.89)	^a^	3.52 (2.41–4.64)	^a^	4.80 (3.59–6.01)	^a^	5.01 (4.24–5.78)	^a^	90.08 (88.43–91.72)	^a^	95.20 (93.99–96.41)	^a^	9.92 (8.28–11.57)	^a^
Female	269 (59.0)	0.19 (0.00–0.82)	^b^	1.63 (0.22–3.04)	^b^	1.84 (0.29–3.38)	^b^	3.64 (2.67–4.61)	^b^	94.54 (92.47–96.61)	^b^	98.16 (96.62–99.71)	^a^	5.46 (3.39–7.53)	^b^
*p*-value		0.002		0.027		0.002		0.02		<0.001		0.002		<0.001	
Age (y)															
≤59	80 (17.5)	1.76 (1.04–2.49)	^a^	4.74 (3.14–6.35)	^a^	6.24 (4.48–8.01)	^a^	4.92 (3.82–6.02)		88.57 (86.21–90.93)	^b^	93.76 (91.99–95.52)	^b^	11.43 (9.07–13.79)	^a^
60–69	144 (31.6)	1.00 (0.43–1.56)	^ab^	3.08 (1.83–4.34)	^ab^	3.94 (2.57–5.30)	^ab^	4.73 (3.87–5.59)		91.19 (89.34–93.03)	^ab^	96.06 (94.70–97.43)	^ab^	8.81 (6.97–10.66)	^ab^
70–79	160 (35.1)	0.34 (0.00–0.92)	^b^	2.13 (0.85–3.42)	^ab^	2.38 (0.99–3.78)	^bc^	4.04 (3.16–4.92)		93.48 (91.60–95.37)	^a^	97.62 (96.22–99.01)	^a^	6.52 (4.63–8.40)	^bc^
≥80	72 (15.8)	0.06 (0.00–0.85)	^b^	0.35 (0.00–2.12)	^b^	0.71 (0.00–2.64)	^c^	3.61 (2.39–4.83)		95.99 (93.38–98.59)	^a^	99.29 (97.36–101.22)	^a^	4.01 (1.41–6.62)	^c^
*p*-value		0.002		0.002		<0.001		0.238		<0.001		<0.001		<0.001	
Period of residence (y)															
≤20	86 (18.9)	0.35 (0.00–1.05)		1.20 (0.00–2.76)	^a^	1.79 (0.10–3.48)	^a^	3.62 (2.55–4.69)		94.83 (92.54–97.12)	^a^	98.21 (96.52–99.90)	^a^	5.17 (2.88–7.46)	^b^
21–40	72 (15.8)	0.37 (0.00–1.15)		2.33 (0.59–4.07)	^a^	2.63 (0.72–4.53)	^a^	3.76 (2.57–4.96)		93.54 (90.99–96.09)	^ab^	97.37 (95.47–99.28)	^ab^	6.46 (3.91–9.01)	^ab^
41–60	156 (34.2)	1.26 (0.69–1.82)		2.58 (1.32–3.84)	^a^	3.80 (2.43–5.17)	^a^	4.98 (4.11–5.84)		91.19 (89.34–93.04)	^b^	96.20 (94.83–97.57)	^ab^	8.81 (6.96–10.66)	^a^
≥61	142 (31.1)	1.19 (0.60–1.79)		4.20 (2.87–5.53)	^a^	5.06 (3.61–6.51)	^a^	4.94 (4.03–5.85)		89.67 (87.72–91.62)	^b^	94.94 (93.49–96.39)	^b^	10.33 (8.38–12.28)	^a^
*p*-value		0.046		0.016		0.014		0.063		0.002		0.014		0.002	
Drinking water															
Groundwater/local drinking water	269 (59.0)	0.99 (0.53–1.46)		2.69 (1.65–3.72)		3.56 (2.44–4.68)		4.72 (4.01–5.43)		91.60 (90.08–93.12)		96.44 (95.32–97.56)		8.40 (6.88–9.92)	
Tap water/purified water	187 (41.0)	0.59 (0.04–1.14)		2.47 (1.25–3.69)		3.07 (1.74–4.40)		3.93 (3.09–4.77)		93.01 (91.22–94.80)		96.93 (95.60–98.26)		6.99 (5.20–8.78)	
*p*-value		0.145		0.723		0.467		0.060		0.117		0.467		0.117	
Seafood intake in the last week (missing *n* = 117)															
Yes	268 (82.5)	0.65 (0.28–1.02)	^a^	1.35 (0.95–1.76)	^a^	2.01 (1.43–2.58)	^a^	4.52 (3.76–5.27)		93.48 (92.34–94.61)		97.99 (97.42–98.57)	^a^	6.52 (5.39–7.66)	^a^
No	57 (17.5)	1.31 (0.68–1.94)	^a^	2.12 (1.42–2.81)	^a^	3.43 (2.44–4.41)	^a^	4.75 (3.46–6.03)		91.83 (89.90–93.76)		96.57 (95.59–97.56)	^b^	8.17 (6.24–10.10)	^a^
*p*-value		0.036		0.028		0.004		0.724		0.087		0.004		0.087	
Distance from mine (km) (missing *n* = 48)															
< 0.5	97 (24.4)	1.14 (0.59–1.70)		4.41 (2.91–5.90)	^a^	5.46 (3.86–7.06)	^a^	5.93 (4.94–6.91)	^a^	88.52 (86.43–90.62)	^a^	94.54 (92.94–96.14)	^b^	11.48 (9.38–13.57)	^a^
0.5–<1.0	97 (24.4)	0.99 (0.41–1.57)		2.71 (1.14–4.29)	^ab^	3.49 (1.79–5.19)	^ab^	3.75 (2.72–4.77)	^b^	92.55 (90.35–94.75)	^ab^	96.51 (94.81–98.21)	^ab^	7.45 (5.25–9.65)	^ab^
1.0–<1.5	95 (23.9)	0.61 (0.00–1.22)		2.87 (1.20–4.53)	^ab^	3.47 (1.70–5.25)	^ab^	3.72 (2.64–4.81)	^b^	92.80 (90.47–95.13)	^b^	96.53 (94.75–98.30)	^ab^	7.20 (4.87–9.53)	^b^
1.5–<3.0	80 (20.1)	0.42 (0.00–1.03)		1.13 (0.00–2.80)	^b^	1.53 (0.00–3.31)	^b^	3.66 (2.57–4.76)	^b^	94.79(92.45–97.13)	^b^	98.47 (96.69–100.25)	^a^	5.21 (2.87–7.55)	^b^
≥3.0	29 (7.3)	0.87 (0.00–1.83)		0.00 (0.00–2.42)	^b^	0.69 (0.00–3.45)	^b^	4.44 (2.74–6.13)	^ab^	94.86(91.23–98.48)	^b^	99.31 (96.55–102.07)	^a^	5.14 (1.52–8.77)	^b^
*p*-value		0.302		0.004		0.002		0.002		<0.001		0.002		<0.001	
As (3+ ), As (5 + ), MMA, and DMA															
1Q (12.60–49.75 μg/g cr)	114 (25.0)	0.55 (0.00–0.16)	^a^	5.90 (4.59–7.20)	^a^	6.45 (5.00–7.90)	^a^	5.73 (4.81–6.65)	^a^	87.82(85.89–89.75)	^c^	93.55 (92.10–95.00)	^c^	12.18 (10.25–14.11)	^a^
2Q (50.27–82.09 μg/g cr)	114 (25.0)	0.29 (0.00–0.92)	^a^	1.60 (0.24–2.95)	^b^	1.89 (0.38–3.39)	^b^	4.45 (3.49–5.41)	^ab^	93.66(91.66–95.67)	^b^	98.11 (96.61–99.62)	^b^	6.34 (4.33–8.34)	^b^
3Q (82.28–124.04 μg/g cr)	114 (25.0)	0.69 (0.07–1.30)	^ab^	1.07 (0.00–2.39)	^b^	1.75 (0.28–3.23)	^b^	3.32 (2.38–4.25)	^b^	94.93(92.97–96.89)	^a^	98.25 (96.77–99.72)	^a^	5.07 (3.11–7.03)	^b^
4Q (125.07–633.97 μg/g cr)	114 (25.0)	1.70 (1.06–2.33)	^b^	0.98 (0.00–2.34)	^b^	2.68 (1.16–4.20)	^b^	3.53 (2.57–4.50)	^b^	93.79(91.77–95.81)	^b^	97.32 (95.80–98.84)	^b^	6.21 (4.19–8.23)	^b^
*p*-value		0.001		<0.001		<0.001		<0.001		<0.001		<0.001		<0.001	

^abc^: Grouping by Bonferroni post-hoc. Estimates with the same letter are not significantly different. Adjusted: least square means adjusted by sex, age, period of residence (missing *n* = 1), drinking water, smoking status, and drinking status.

## Data Availability

Restrictions apply to the availability of these data. Data were obtained from the Korea National Institute of Environmental Research and are available at https://www.nier.go.kr/NIER/egovEngIndex.jsp (accessed on 3 March 2021) with the permission of the Korea National Institute of Environmental Research.

## Supplementary Materials

The following are available online at https://www.mdpi.com/article/10.3390/toxics9060138/s1. Figure S1: Distribution of As species concentrations by AsIII, AsV, MMA, and DMA percentiles. Table S1: Adjusted geometric mean concentrations of As species in 102 abandoned metal mines. Table S2: Correlation coefficient of As species concentration and proportion.

## Abbreviation

AM: arithmetic mean; AMS: The Health Effects Survey of Abandoned Metal Mines; As: arsenic; AsIII: arsenite; AsV: arsenate; ATSDR: Agency for Toxic Substances and Disease Registry; cAs: chemical species of arsenic; CCC: concordance correlation coefficient; CI: confidence interval; DMA: dimethylarsenic acid; GM: geometric mean; hAs: hazardous arsenic species; inorganic-related As species; AsIII, AsV, MMA, and DMA; hAs_AAS_: hazardous arsenic species concentration by hydride generation atomic absorption spectroscopy; hAs_ICP_: hazardous arsenic species concentration by high-performance liquid chromatography and inductively coupled plasma-mass spectrometry; HG-AAS: hydride generation atomic absorption spectroscopy; HPLC-ICP-MS: high-performance liquid chromatography and inductively coupled plasma-mass spectrometry; iAs: inorganic arsenic; KMOE: Korea Ministry of Environment; KNHAENS: Korea National Health and Nutrition Examination Survey; KNIRE: Korea National Institute of Environmental Research; LSMs: least square means; MMA: monomethylarsinic acid; NHANES: U.S. National Health and Nutrition Examination Survey; NPL: National Priorities List; oAs: organic arsenic; PLHS: priority list of hazardous substances; SD: standard deviation; tAs: total arsenic; UAL: urinary arsenic concentration; UAL_AAS_: urinary arsenic concentration by hydride generation atomic absorption spectroscopy; UAL_ICP_: urinary arsenic concentration by high-performance liquid chromatography and inductively coupled plasma-mass spectrometry.
