# Supplementary material for: Necessity of Assessing Biological Exposure to Arsenic Species by Two Representative Analytical Methods
Source: Toxics. 2021 Jun 11;9(6):138. doi: 10.3390/toxics9060138 (PMC8230710; doi:10.3390/toxics9060138)
Supplement: Supplementary file 1 [file toxics-09-00138-s001.zip › toxics-1150140-SI.pdf]

toxics

MDPI

Article

Supplementary Materials: Necessity of Assessing Biological Exposure to Arsenic Species by Two Representative Analytical Methods

Jeong-Wook Seo and Young-Seoub Hong

Table S1. Adjusted geometric mean concentrations of As species in 102 abandoned metal mines.

	n (%)	Adjusted GM (95% CI) (µg/g cr)							
		Inorganic As			Organic As			AsIII + AsV + MMA + DMA	HG-AAS
		AsIII	AsV	Subtotal	MMA	DMA	Subtotal		
**Total**	442 (100.0)	0.10 (0.08–0.13)	0.31 (0.24–0.39)	0.57 (0.45–0.72)	0.90 (0.65–1.23)	68.75 (61.61–76.73)	72.19 (64.85–80.37)	74.85 (67.50–82.99)	55.07 (48.41–62.64)
Sex									
Male	182 (41.2)	0.13 (0.10–0.17) [a]	0.35 (0.26–0.47)	0.72 (0.55–0.94) [a]	1.09 (0.76–1.58)	59.05 (51.95–67.13) [a]	62.50 (55.14–70.84) [a]	65.98 (58.48–74.43) [a]	44.74 (38.49–52.01) [a]
Female	260 (58.8)	0.08 (0.05–0.11) [b]	0.27 (0.18–0.39)	0.46 (0.32–0.64) [b]	0.74 (0.46–1.18)	80.05 (68.02–94.21) [b]	83.40 (71.12–97.79) [b]	84.91 (72.85–98.98) [b]	67.77 (55.98–82.06) [b]
p-value		0.008	0.256	0.030	0.168	0.002	0.003	0.007	<0.001
Age (y)									
≤59	77 (17.4)	0.16 (0.11–0.24) [a]	0.48 (0.31–0.74) [a]	1.06 (0.72–1.58) [a]	0.80 (0.47–1.37)	49.77 (41.31–59.96) [b]	52.68 (43.91–63.20) [b]	56.48 (47.40–67.31) [b]	39.62 (31.83–49.31) [b]
60–69	140 (31.7)	0.10 (0.08–0.14) [a]	0.36 (0.26–0.50) [a]	0.65 (0.48–0.88) [ab]	0.98 (0.65–1.48)	62.46 (54.08–72.14) [ab]	65.96 (57.30–75.94) [ab]	68.89 (60.15–78.90) [ab]	47.48 (40.08–56.23) [ab]
70–79	154 (34.8)	0.08 (0.06–0.11) [a]	0.23 (0.16–0.32) [a]	0.39 (0.28–0.53) [b]	0.82 (0.54–1.25)	76.59 (66.10–88.75) [a]	80.02 (69.29–92.42) [a]	82.12 (71.49–94.33) [a]	60.84 (51.17–72.33) [a]
≥80	71 (16.1)	0.08 (0.05–0.12) [a]	0.22 (0.14–0.35) [a]	0.40 (0.26–0.61) [b]	1.01 (0.56–1.81)	93.84 (76.56–115.04) [a]	97.69 (80.06–119.20) [a]	98.23 (81.10–118.98) [a]	80.36 (63.27–102.07) [a]
p-value		0.018	0.013	<0.001	0.824	<0.001	<0.001	<0.001	<0.001
Period of residence (y)									
≤20	85 (19.2)	0.09 (0.06–0.13) [ab]	0.21 (0.14–0.32)	0.38 (0.26–0.55) [b]	0.55 (0.33–0.92) [b]	69.73 (58.31–83.39)	72.65 (61.00–86.53)	73.95 (62.50–87.51)	61.54 (49.88–75.93) [a]
21–40	69 (15.6)	0.07 (0.05–0.11) [b]	0.32 (0.20–0.51)	0.49 (0.32–0.74) [ab]	0.70 (0.39–1.25) [ab]	74.46 (60.89–91.06)	77.65 (63.79–94.53)	79.83 (66.06–96.48)	59.22 (46.76–75.01) [a]

41–60	152 (34.4)	0.14 (0.11–0.20) [a]	0.30 (0.21–0.42)	0.72 (0.53–0.98) [a]	1.41 (0.93–2.14) [a]	72.69 (62.90–84.00)	76.94 (66.80–88.63)	80.19 (69.99–91.89)	56.62 (47.77–67.11) [a]
≥61	136 (30.8)	0.11 (0.08–0.15) [a]	0.43 (0.30–0.61)	0.81 (0.59–1.12) [a]	1.20 (0.77–1.86) [a]	59.21 (50.80–69.00)	62.58 (53.89–72.69)	66.29 (57.39–76.56)	44.56 (37.23–53.34) [a]
p-value		0.013	0.053	0.004	0.006	0.120	0.116	0.151	0.049
Drinking water									
Ground water/local drinking water	259 (58.6)	0.11 (0.09–0.14)	0.31 (0.23–0.41)	0.63 (0.49–0.81)	0.99 (0.71–1.40)	65.16 (57.90–73.34)	68.68 (61.19–77.09)	71.40 (63.88–79.80)	53.82 (46.84–61.83)
Tap water/purified water	183 (41.4)	0.09 (0.07–0.12)	0.30 (0.22–0.42)	0.52 (0.39–0.70)	0.81 (0.54–1.21)	72.54 (63.03–83.49)	75.89 (66.15–87.06)	78.47 (68.75–89.56)	56.35 (47.78–66.46)
p-value		0.119	0.916	0.207	0.308	0.129	0.149	0.156	0.580
Seafood intake in the last week (missing *n* = 117)									
Yes	268 (82.5)	0.09 (0.07–0.12)	0.26 (0.20–0.33) [a]	0.48 (0.38–0.61) [a]	1.05 (0.71–1.55)	69.31 (61.23–78.45)	72.83 (64.50–82.23)	74.36 (65.97–83.82)	56.86 (50.24–64.35)
No	57 (17.5)	0.14 (0.09–0.23)	0.42 (0.27–0.64) [a]	0.85 (0.57–1.28) [a]	0.79 (0.40–1.55)	64.99 (52.60–80.29)	68.53 (55.70–84.30)	71.04 (57.91–87.15)	52.49 (42.50–64.83)
p-value		0.085	0.022	0.005	0.401	0.542	0.555	0.653	0.448
Distance from mine (km) (missing *n* = 48)									
< 0.5	95 (24.1)	0.12 (0.08–0.17)	0.52 (0.36–0.76) [a]	0.96 (0.68–1.36) [a]	1.31 (0.81–2.12)	52.00 (44.04–61.39) [bc]	55.65 (47.29–65.49) [b]	59.08 (50.50–69.11) [c]	38.95 (32.16–47.16) [b]
0.5–< 1.0	95 (24.1)	0.10 (0.07–0.14)	0.36 (0.24–0.53) [ab]	0.66 (0.46–0.95) [ab]	0.86 (0.52–1.44)	76.36 (64.02–91.07) [b]	79.59 (66.97–94.60) [b]	82.65 (69.98–97.62) [b]	60.51 (49.39–74.12) [ab]
1.0– < 1.5	95 (24.1)	0.09 (0.06–0.14)	0.32 (0.21–0.49) [abc]	0.56 (0.38–0.83) [ab]	0.78 (0.46–1.32)	76.12 (63.32–91.51) [b]	79.39 (66.28–95.09) [b]	82.62 (69.43–98.31) [b]	62.44 (50.51–77.20) [ab]
1.5– < 3.0	80 (20.3)	0.08 (0.06–0.12)	0.21 (0.14–0.33) [bc]	0.40 (0.27–0.58) [b]	0.73 (0.43–1.24)	81.41 (67.67–97.94) [a]	84.76 (70.71–101.60) [a]	86.06 (72.27–102.48) [a]	71.29 (57.61–88.21) [a]
≥3.0	29 (7.4)	0.09 (0.05–0.16)	0.11 (0.06–0.22) [c]	0.28 (0.15–0.51) [b]	0.60 (0.26–1.38)	49.14 (36.90–65.44) [c]	51.59 (38.96–68.32) [c]	51.86 (39.57–67.98) [d]	46.22 (33.23–64.29) [ab]
p-value		0.587	<0.001	<0.001	0.287	<0.001	<0.001	<0.001	<0.001
As (3+) + As (5+) + MMA + DMA									
1Q (12.60–49.27 μg/g cr)	111 (25.1)	0.08 (0.06–0.11) [a]	0.46 (0.32–0.67) [a]	0.74 (0.53–1.04)	0.74 (0.47–1.17)	26.72 (25.00–28.56) [c]	28.67 (26.88–30.58) [c]	30.96 (29.14–32.90) [c]	22.20 (19.65–25.08) [c]

2Q (49.34–82.17 µg/g cr)	111 (25.1)	0.10 (0.07–0.14) a	0.30 (0.21–0.45) ab	0.57 (0.40–0.81)	1.10 (0.68–1.76)	59.90 (55.91–64.19) b	63.04 (58.96–67.40) b	64.39 (60.45–68.59) b	45.98 (40.50–52.19) b
3Q (80.33–120.28 µg/g cr)	110 (24.9)	0.09 (0.07–0.13) a	0.23 (0.16–0.34) b	0.43 (0.31–0.60)	1.06 (0.67–1.67)	92.35 (86.38–98.73) b	95.90 (89.89–102.32) b	98.16 (92.34–104.34) b	74.66 (66.04–84.41) b
4Q (120.85–633.97 µg/g cr)	110 (24.9)	0.14 (0.10–0.20) a	0.25 (0.17–0.36) ab	0.57 (0.40–0.81)	0.79 (0.49–1.26)	170.48 (159.15–182.62) a	176.29 (164.92–188.44) a	179.01 (168.10–190.62) a	134.45 (118.50–152.55) a
p-value		0.050	0.014	0.075	0.309	<0.001	<0.001	<0.001	<0.001

abc: Grouping by Bonferroni post-hoc. Estimates with the same letter are not significantly different. Adjusted: least square means adjusted by sex, age, period of residence (missing n = 1), drinking water, smoking status, and drinking status.

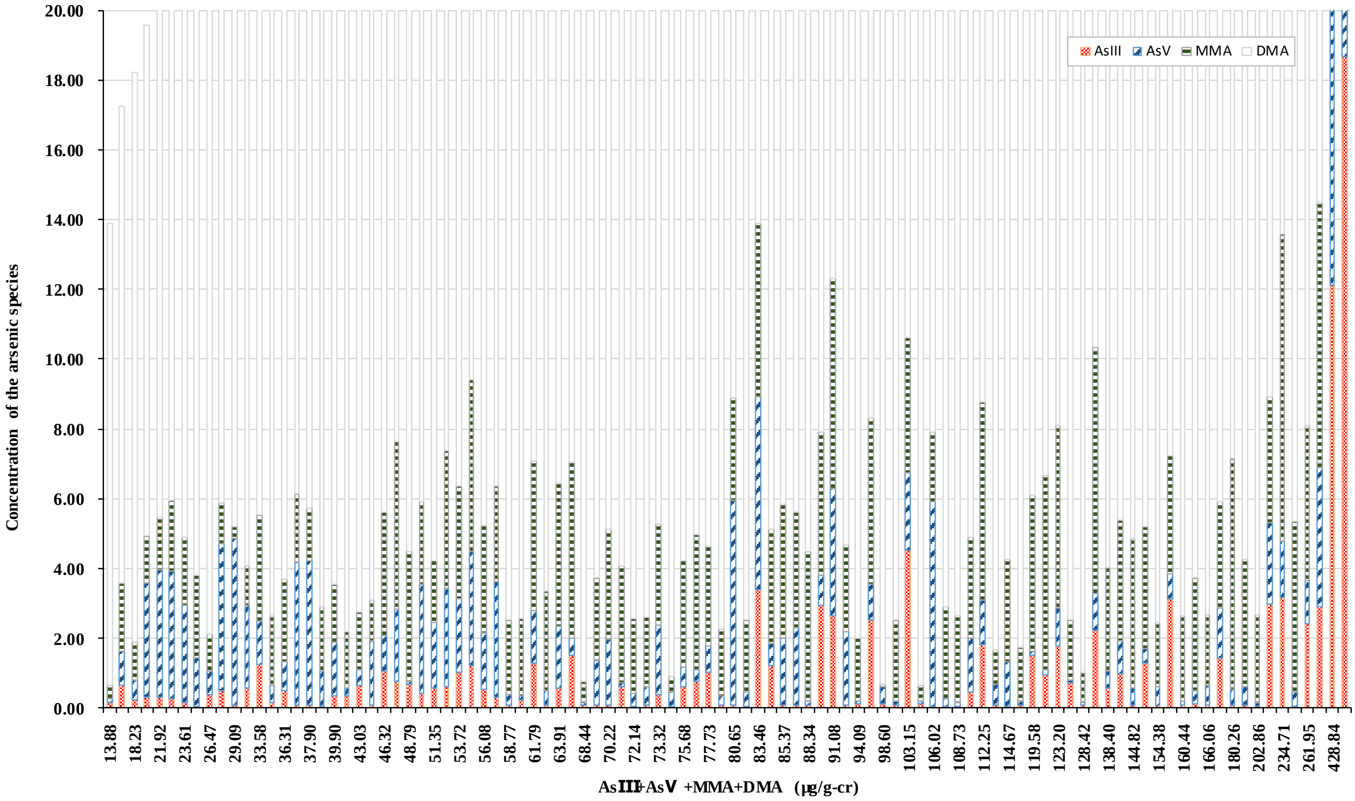

Figure S1. Distribution of As species concentrations by AsIII + AsV + MMA + DMA percentile.

Table S2. Correlation coefficient of As species concentration and proportion.

Group	As Species	HPLC-ICP-MS							HG-AAS	Proportion of As Species Relative to the Sum of Concentrations of 4 As Species						
		Inorganic As			Organic As			Sum of 4 As Species		Inorganic As			Organic As			Sum of 3 As Species
		AsIII	AsV	Sub-total	MMA	DMA	Subtotal			AsIII	AsV	Subtotal	MMA	DMA	Subtotal	

103 mines (n = 457)	HPLC-ICP-MS	Inorganic As	AsIII	1	0.549	0.919	0.822	0.313	0.384	0.479	0.458	**0.763**	0.049	0.350	0.328	-0.410	-0.350	0.410
			AsV		1	0.835	0.681	0.208	0.269	0.360	0.234	0.328	**0.524**	0.599	0.293	-0.579	-0.599	0.579
			Subtotal			1	0.864	0.304	0.381	0.485	0.412	0.658	0.280	**0.514**	0.355	-0.544	-0.514	0.544
		Organic As	MMA				1	0.358	0.446	0.530	0.436	0.527	0.062	0.267	**0.545**	-0.447	-0.267	0.447
			DMA					1	0.995	0.979	0.891	0.079	-0.235	-0.179	-0.188	**0.219**	0.179	-0.219
			Subtotal						1	0.993	0.899	0.130	-0.219	-0.144	-0.124	0.164	**0.144**	-0.164
		Sum of 4 As species								1	0.902	0.205	-0.172	-0.071	-0.073	0.086	0.071	-0.086
	HG-AAS										1	0.207	-0.230	-0.122	-0.120	0.145	0.122	-0.145
	Proportion of As species relative to the sum of concentrations of 4 As species	Inorganic As	AsIII									1	0.057	0.453	0.437	-0.536	-0.453	0.536
			AsV										1	0.916	0.199	-0.772	-0.916	0.772
			Subtotal											1	0.353	-0.905	-1.000	0.905
		Organic As	MMA												1	-0.718	-0.353	0.718
			DMA													1	0.905	-1.000
			Subtotal														1	-0.905
		Sum of 3 AS species																1
